# Above-ground biomass and structure of 260 African tropical forests

**DOI:** 10.1098/rstb.2012.0295

**Published:** 2013-09-05

**Authors:** Simon L. Lewis, Bonaventure Sonké, Terry Sunderland, Serge K. Begne, Gabriela Lopez-Gonzalez, Geertje M. F. van der Heijden, Oliver L. Phillips, Kofi Affum-Baffoe, Timothy R. Baker, Lindsay Banin, Jean-François Bastin, Hans Beeckman, Pascal Boeckx, Jan Bogaert, Charles De Cannière, Eric Chezeaux, Connie J. Clark, Murray Collins, Gloria Djagbletey, Marie Noël K. Djuikouo, Vincent Droissart, Jean-Louis Doucet, Cornielle E. N. Ewango, Sophie Fauset, Ted R. Feldpausch, Ernest G. Foli, Jean-François Gillet, Alan C. Hamilton, David J. Harris, Terese B. Hart, Thales de Haulleville, Annette Hladik, Koen Hufkens, Dries Huygens, Philippe Jeanmart, Kathryn J. Jeffery, Elizabeth Kearsley, Miguel E. Leal, Jon Lloyd, Jon C. Lovett, Jean-Remy Makana, Yadvinder Malhi, Andrew R. Marshall, Lucas Ojo, Kelvin S.-H. Peh, Georgia Pickavance, John R. Poulsen, Jan M. Reitsma, Douglas Sheil, Murielle Simo, Kathy Steppe, Hermann E. Taedoumg, Joey Talbot, James R. D. Taplin, David Taylor, Sean C. Thomas, Benjamin Toirambe, Hans Verbeeck, Jason Vleminckx, Lee J. T. White, Simon Willcock, Hannsjorg Woell, Lise Zemagho

**Affiliations:** 1 Department of Geography, University College London, London WC1E 6BT, UK; 2 School of Geography, University of Leeds, Leeds LS2 9JT, UK; 3 Plant Systematic and Ecology Laboratory, Department of Biology, Higher Teachers' Training College, University of Yaounde I, PO Box 047, Yaounde, Cameroon; 4 Center for International Forestry Research, Bogor, Indonesia; 5 University of Wisconsin-Milwaukee, PO Box 413, Milwaukee, WI 53201, USA; 6 Smithsonian Tropical Research Institute, Apartado Postal 0843-03092, Panama; 7 Mensuration Unit, Forestry Commission of Ghana, Kumasi, Ghana; 8 Centre for Ecology and Hydrology, Bush Estate, Penicuik, Midlothian EH26 0QB, UK; 9 Landscape Ecology and Vegetal Production Systems Unit, Université Libre de Bruxelles, Brussels, Belgium; 10 Biodiversity and Landscape Unit, Gembloux Agro-Bio Tech, Université de Liège, Gembloux, Belgium; 11 Ecole Régionale post-universitaire d'Aménagement et de gestion Intégrés des Forêts et Territoires tropicaux, Kinshasa, Republic Democratic of Congo; 12 Laboratory for Wood Biology and Xylarium, Royal Museum for Central Africa, Tervuren, Belgium; 13 Isotope Bioscience Laboratory-ISOFYS, Department of Applied Analytical and Physical Chemistry, Faculty of Bioscience Engineering, Ghent University, Ghent, Belgium; 14 Rougier-Gabon, Oloumi Industrial Estate, PO Box 130, Libreville, Gabon; 15 Nicholas School of the Environment, Duke University, PO Box 90328, Durham, NC 27708, USA; 16 Grantham Research Institute on Climate Change and the Environment, London School of Economics, Tower 3, Clements Inn Passage, London WC2A 2AZ, UK; 17 Forestry Research Institute of Ghana (FORIG), UP Box 63, KNUST, Kumasi, Ghana; 18 Department of Botany and Plant Physiology, Faculty of Science, University of Buea, PO Box 63 Buea-Cameroon; 19 Institut de Recherche pour le Développement (IRD), Unité Mixte de Recherche AMAP (Botanique et Bioinformatique de l'Architecture des Plantes), Boulevard de la Lironde, Montpellier, France; 20 Laboratory of Tropical and Subtropical Forest Regions, Unit of Forest and Nature Management, University of Liège, Gembloux, Belgium; 21 Nature +, c/o Gembloux Agro-Bio Tech, University of Liège, Gembloux, Belgium; 22 Wildlife Conservation Society-DR Congo, PO Box 240, Kinshasa I, DR Congo; 23 Centre de Formation et de Recherche en Conservation Forestiere (CEFRECOF), Democratic Republic of Congo; 24 128 Busbridge Lane, Godalming, Surrey GU7 1QJ, UK; 25 Royal Botanic Garden Edinburgh, 20A Inverleith Row, Edinburgh EH3 5LR, UK; 26 Lukuru Wildlife Research Foundation, Kinshasa, Gombe, Democratic Republic of Congo; 27 Division of Vertebrate Zoology, Yale Peabody Museum of Natural History, New Haven, CT, USA; 28 Département Hommes Natures Sociétés, Muséum national d'histoire naturelle, Brunoy, France; 29 Institute of Agricultural Engineering and Soil Science, Faculty of Agricultural Sciences, Universidad Austral de Chile, Valdivia, Chile; 30 Precious Woods Gabon, Libreville, Gabon; 31 Agence Nationale des Parcs Nationaux, BP 20379, Libreville, Gabon; 32 Institut de Recherche en Écologie Tropicale, BP 13354 Libreville, Gabon; 33 School of Natural Sciences, University of Stirling, Stirling FK9 4LA, UK; 34 Laboratory of Plant Ecology, Department of Applied Ecology and Environmental Biology, Faculty of Bioscience Engineering, Ghent University, Ghent, Belgium; 35 Wildlife Conservation Society, PO Box 7487, Kampala, Uganda; 36 School of Earth and Environmental Science, James Cook University, Cairns, Australia; 37 School of Geography and the Environment, University of Oxford, Oxford OX1 3QY, UK; 38 CIRCLE, Environment Department, University of York, York YO10 5DD, UK; 39 Flamingo Land Ltd, Kirby Misperton, North Yorkshire YO17 6UX, UK; 40 Department of Environmental Management and Toxicology, University of Agriculture, PMB 2240, Abeokuta, Ogun, Nigeria; 41 Department of Zoology, University of Cambridge, Cambridge CB2 3EJ, UK; 42 Bureau Waardenburg bv, Postbus 365, Culemborg AJ 4100, The Netherlands; 43 School of Environment, Science, and Engineering, Southern Cross University, Lismore, New South Wales 2480, Australia; 44 Institute of Tropical Forest Conservation, PO Box 44, Kabale, Uganda; 45 Forum for the Future, Overseas House, 19-23 Ironmonger Row, London EC1V 3QN, UK; 46 Department of Geography, National University of Singapore, Singapore 119615, Republic of Singapore; 47 Faculty of Forestry, University of Toronto, 33 Willcocks Street, Toronto, Ontario, CanadaM5S 3B3; 48 Service Evolution Biologique et Ecologie, Faculté des Sciences, Université Libre de Bruxelles, Brussels, Belgium; 49 Department of Life Sciences, University of Southampton, Southampton SO17 1BJ, UK; 50 Sommersbergseestrasse, 291, Bad Aussee 8990, Austria

**Keywords:** climate, soil, wood density, Congo Basin, east Africa, west Africa

## Abstract

We report above-ground biomass (AGB), basal area, stem density and wood mass density estimates from 260 sample plots (mean size: 1.2 ha) in intact closed-canopy tropical forests across 12 African countries. Mean AGB is 395.7 Mg dry mass ha^−1^ (95% CI: 14.3), substantially higher than Amazonian values, with the Congo Basin and contiguous forest region attaining AGB values (429 Mg ha^−1^) similar to those of Bornean forests, and significantly greater than East or West African forests. AGB therefore appears generally higher in palaeo- compared with neotropical forests. However, mean stem density is low (426 ± 11 stems ha^−1^ greater than or equal to 100 mm diameter) compared with both Amazonian and Bornean forests (cf. approx. 600) and is the signature structural feature of African tropical forests. While spatial autocorrelation complicates analyses, AGB shows a positive relationship with rainfall in the driest nine months of the year, and an opposite association with the wettest three months of the year; a negative relationship with temperature; positive relationship with clay-rich soils; and negative relationships with C : N ratio (suggesting a positive soil phosphorus–AGB relationship), and soil fertility computed as the sum of base cations. The results indicate that AGB is mediated by both climate and soils, and suggest that the AGB of African closed-canopy tropical forests may be particularly sensitive to future precipitation and temperature changes.

## 1. Introduction

Comparative studies of the above-ground biomass (AGB) of tropical forests exist for South America [1–3] and Asia [4] but not for Africa. Thus, some ostensibly simple questions remain unanswered: how much AGB does an average structurally intact African tropical forest store? Where in Africa is biomass lower or higher; and what controls this spatial variation? How do African forest AGB values compare with those on other continents? Here, we collate standardized AGB data from across tropical Africa to provide a first answer to these broad questions.

Understanding the spatial patterns of biomass in African forests is important on at least four counts. First, to provide insights into how tropical forests function. Africa provides a useful contrast with Amazonia in terms of separating possible causal factors underlying AGB variation, as unlike Amazonia, Africa does not possess a strong east–west gradient in soil fertility that coincides with other gradients such as mean annual air temperature [1,3,5]. Therefore, studying African forests may assist in developing a more coherent understanding of tropical biomass variation and the relative contributions of climate, soils and disturbance. Additionally, recent work suggests some systematic neo- versus palaeotropical differences in forest structure (i.e. South American versus Africa/Asia forests; [6]), and perhaps AGB varies similarly, as some recent analyses suggest [7]. Second, biomass estimates provide information on ‘emissions factors’ for estimating carbon losses from deforestation and forest degradation [8]. Third, they can assist calibrating and validating carbon mapping exercises [9]. Fourth, modelling tropical forests requires data to both develop and test representations of African forests and their response to a changing environment [10].

The live biomass density of a tropical forest is the sum of the biomass of all living organisms per unit area. This is determined by both the rate of fixation of carbon into root, stem, branch and leaf material per unit area, and how long that fixed material is resident as living mass in each of those biomass pools. Hence, both the net primary productivity (NPP) and the biomass residence time (**τ**_W_, 1/biomass turnover rate) determine a forests’ AGB. In practice, for old-growth forests the turnover times of fine root and leaf material are much shorter (approx. 1–2 years) than that of woody biomass (approx. 50–100 years), and hence total AGB is almost entirely determined by the rate of production of woody biomass (NPP_WOOD_; some 20–40% of NPP [11]) and its residence time. Thus, all other things being equal, a forest with higher NPP_WOOD_ should have greater AGB. Similarly, a forest with a greater **τ**_W_ will accumulate NPP_WOOD_ over more years, leading to greater AGB. Thus, *a priori*, resource availability should affect AGB via NPP_WOOD_, and the size–frequency distribution of disturbance events should affect AGB via **τ**_w_. These disturbance events may be endogenous, for example, related to species life-history traits, soil physical characteristics or biotic interactions (from plant disease to foraging elephants), or exogenous, for example via climatic extremes, or some combination of the two. A third possible class of effect is associated with the species pool available in a given forest that may systematically elevate or depress AGB via effects on either NPP_WOOD_ or **τ**_w_. This may be important given evidence of the relationship between geology and tree species distributions [12,13], and contribute to the high AGB in Southeast Asian forests dominated by Dipterocarpaceae [4,6]. These factors may be nonlinear (soil depth beyond a certain level may have no effect on **τ**_w_), co-correlated (precipitation and soil fertility [14]) or interacting (species growing on high-fertility soils may have shorter lifespans, shortening **τ**_w_ [3]). A recent evaluation of Amazonian AGB patterns highlights the complexity of explaining spatial patterns of AGB variation [3].

The evidence for the effects of individual drivers of spatial differences in AGB within tropical forests is limited, but allows hypotheses to be articulated. Each forest grows on a particular soil under a particular climatic regime. In terms of climate, theory suggests that AGB will be lower when NPP is reduced in forests experiencing a dry season where growth is reduced or ceases owing to a limit in water availability, as has been documented [1,2,4]. Although when accounting for the spatial autocorrelation, this effect on NPP appeared much reduced for Amazon forests [3]. Conversely, extremely wet forests have lower AGB than moist forests [15], perhaps attributable to a lower NPP owing to the cloudiness associated with high rainfall reducing incoming insolation rates [14,16,17]. Hence, high wet-season rainfall may be associated with low AGB. However, simple wet/dry season comparisons are more complex in Africa as the movement of the intertropical convergence zone generates two wet and two dry seasons annually over much of Central Africa, and tropical forests across Africa are on average drier than those in the Americas of Asia [18].

Low air temperature may restrict the efficiency of photosynthesis, hence higher air temperatures in the coolest part of the year may be associated with higher AGB. By contrast, forests growing under higher air temperatures may have higher respiration costs, and if photosynthesis is not higher (or reduced because of higher atmospheric water vapour pressure deficits [19]), NPP may be lower and hence AGB—other things being equal—would be lower. Therefore, forests growing under very high air temperature may be generally associated with a lower AGB. Although Amazonian AGB was not significantly related to mean annual air temperature, wood production was, however, negatively associated with it [3], and in Asia most of the best models relating AGB to environmental conditions do not include temperature [4], suggesting any AGB–temperature relationship may be relatively weak, or is being masked by other covarying factors. We therefore consider both temperature and precipitation as potential drivers of spatial variation in AGB.

The impact of soils on AGB is likely to be complex. Developmentally older soils tend to provide fewer of the nutrients plants require than do younger soils, and hence are poorer substrates for plant growth, but conversely are often deeper and structurally provide improved water retention, and hence are better for plant growth and biomass support [5,14]. Thus, a separation of plant-relevant soil physical and chemical characteristics is necessary to disentangle the likely opposing impacts of nutrient availability on AGB via NPP_WOOD_ and physical soil characteristics via **τ**_w_. Additionally, it is uncertain whether it is phosphorus and/or other nutrients that are the most important fertility-related soil parameters affecting NPP_WOOD_. Furthermore, soil data are often unavailable for forest inventory plots, and methods of soil analysis may also be different: all of which complicate analyses of soil effects on tropical forest function. Based on available evidence, we predict structurally poor soils, including coarse-textured sandy soils, to be associated with lower AGB. The predicted response to the higher availability of soil nutrients is ambiguous, as NPP_WOOD_ is likely to be higher, hence higher AGB might be expected, yet such forest stands may become dominated by species with low wood mass density (WMD) which tend to have shorter lifespans (shorter **τ**_W_), and hence a lower AGB. Positive AGB–nutrient relationships from Borneo imply the increase in NPP_WOOD_ dominates there [4], whereas in Amazonia, the decline in **τ**_W_ appears to dominate [1,3]. A Central African study suggests that higher NPP_WOOD_ and lower **τ**_W_ likely balance each other in terms of their impact on AGB [20].

The role of exogenous disturbance events in determining AGB is also difficult because such events are difficult to characterize *ex posto facto*. However, we may get insights in three ways. First, stem density provides insights as low disturbance rates over preceding decades are likely to result in greater biomass allocated to fewer stems, because when exogenous disturbance events are rare, larger older trees should dominate, shading out and thus reducing the growth rates and survival probability of smaller trees (‘self thinning’). Second, habitat fragmentation may elevate disturbance rates, altering AGB patterns in remaining forest [21]. Third, community-average WMD should be lower in more frequently disturbed and hence dynamic forests comprising greater numbers of earlier successional species [22]. Therefore, we report on all of AGB, basal area (BA), stand WMD and stem density for our 260 forest monitoring plots encompassing West, Central and East Africa, also investigating their relationship with soil, climate and fragmentation variables. Analytically, we use a series of statistical techniques to attempt to build a synthetic understanding of the likely controls on forest AGB across tropical Africa.

## 2. Methods

### (a) Data collection and processing

Forest inventory plot data, collected and collated as part of the African Tropical Rainforest Observatory Network (AfriTRON; www.afritron.org), were selected for analysis when conforming to the following criteria: closed-canopy tropical forest; geo-referenced; all trees greater than or equal to 100 mm diameter measured; greater than or equal to 0.2 ha; majority of stems identified to species; old-growth and structurally intact, i.e. not impacted by recent selective logging or fire; mean annual air temperature greater than or equal to 20°C and greater than or equal to 1000 mm mean annual precipitation (from WorldClim [23]). Three remaining plots previously characterized by researchers as ‘montane’ forest were excluded. In all plots, tree diameter was measured at 1.3 m along the stem from the ground, or above buttresses, if present. The 260 plots (total, 312.5 ha) that conformed to the criteria comprised 132 899 stems, of which 85% were identified to species and 96% to genera. Further details are given in the electronic supplementary material.

**Figure 1.**
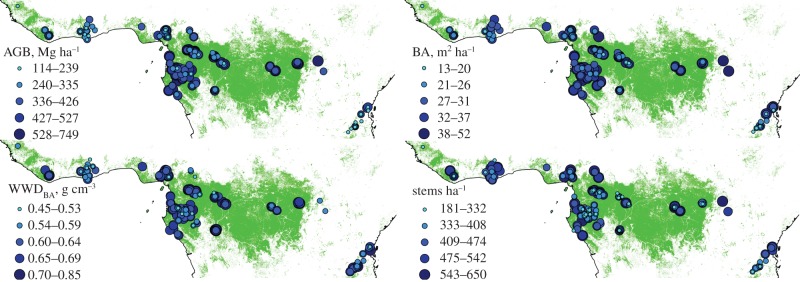
Above-ground biomass (AGB), basal area (BA), basal area-weighted wood mass density (WMD_BA_), and stem density for 260 plots in closed-canopy tropical forest. Green represents ‘closed forest’ and ‘flooded forest’ categories from the 300 m resolution European Space Agency Globcover (v. 2.3) map for the year 2009. (Online version in colour.)

For each plot, we calculated (i) stem density greater than or equal to 100 mm diameter per ha; (ii) the BA (sum of the cross-sectional area at 1.3 m, or above buttresses, of all live trees) in m^2^ ha^−1^; (iii) BA-weighted wood mass density (WMD_BA_), i.e. the mean of the WMD of each stem weighted by its BA, where WMD is dry mass/fresh volume in g cm^−3^. The best taxonomic match wood density of each stem was extracted from a global database [24,25] following a well-established procedure [26]; (iv) AGB (including stem, branches and leaves) was calculated using the Chave *et al*. [15] ‘moist forest’ equation to estimate the AGB of each tree in the plot, using diameter, WMD and tree height, with height estimated from diameter using the recommended regional equations for West (region west of the Dahomey gap), Central (Congo–Ogouée Basin and contiguous forest) and East (east of Congo Basin) Africa, as defined in [7], and expressed dry mass as Mg ha^−1^ (= metric tonnes ha^−1^). The stem density BA, WMD, WMD_BA_ and AGB values were calculated using the http://www.forestplots.net/ data management facility [27]; version 13 April 2013 [28]. The locations of the study plots are shown in figure 1.

Average mean annual temperature (*T*_A_), mean monthly maximum air temperature (*T*_max_), mean monthly minimum air temperature (*T*_min_), mean temperature in the warmest and coldest quarters (*T*_WARMQ_, *T*_COLDQ_), temperature seasonality (coefficient of variation; *T*_CV_) and average mean annual precipitation (*P*_A_), mean monthly maximum precipitation (*P*_max_), mean monthly minimum precipitation (*P*_min_), precipitation in the wettest and driest quarters (*P*_WETQ_, *P*_DRYQ_) and precipitation seasonality (coefficient of variation; *P*_CV_) were extracted from the WorldClim database at the finest resolution available (30′; [23]), giving mean long-term climate data (approx. 1950–2000) for each plot location (see the electronic supplementary material for further details).

Detailed information on soils was not available for most plots, but the soil class or type was often known or estimated from data outside the plot, local knowledge, local soil or geology [29]. For each plot, we therefore had a notional soil type, and where necessary this information was converted to a standard classification and soil variables extracted (for 0–30 cm and 30–100 cm depth) for the corresponding soil type at or closest to the plot location from the FAO Digital Soil Map of the World dataset [29]. This provides a method of incorporating consistent soil information, while avoiding the possible problem of incorrectly assigning plots overlying non-dominant soil types, or averaging data from plots on differing soil types within the same interpolated soil map grid square. Hence, plots within the same landscape on differing soil types are assigned corresponding differing soil parameters. The soil data are to be treated with caution, as they are not *in situ* data, particularly as soil geographers sometimes use vegetation characteristics themselves as an aid to their mapping of soil [30], giving rise to a potential tautology. Nevertheless, our approach taken here incorporates the *in situ* data available and avoids some common pitfalls of using gridded soil data allowing for a first-order analysis of any likely edaphic effects on the studied stand properties.

To test for soil-related effects, we used (i) principal components analysis (PCA) on the soil-structure-related data (0–100 cm), giving a sand–clay axis (PC1 sand; low values are high sand content) and a silt axis (PC2 clay–silt; high values are clay-rich, low values silt-rich; loadings in the electronic supplementary material); (ii) sum of exchangeable bases (0–30 cm), in cmol kg^−1^ (∑B), the most relevant to tree growth cation-related plant nutrition variable in the FAO dataset; (iii) C : N ratios as a surrogate for plant available phosphorus. Phosphorus availability is likely to be very important for tree growth but is not reported in the FAO or other large-scale soil datasets. However, soil C : N ratio (0–30 cm) has been shown to be strongly negatively correlated with total extractable phosphorus across in Amazonia [5], and unpublished African *in situ* soil data also support this notion (S. Lewis *et al*., unpublished data). Additionally, we also define soil classes based on pedogenic development, following the scheme in reference [31]: all soils younger than alisols (in this dataset cambisols and histosols), score 1; all soils younger than ferralsols but older than alisols, score 2; all ferralsols, score 3.

Habitat fragmentation indices were devised using Google Earth Pro. We measured the distance from the plot centre to (i) the nearest forest edge (any absence of forest cover greater than or equal to 1 ha), giving a distance to edge (fragment edge in km, *F*_E_) and (ii) the nearest edge of a clearing  greater than or equal to 1 ha in eight directions every 45° from north, from which we estimated fragment size by summing the areas of the eight triangles generated (fragment area in km^2^; *F*_A_).

### (b) Statistical analysis

The dataset is complex with explanatory variables spatially autocorrelated. Furthermore, some of the soil types are rare, and temperature- and precipitation-related variables also correlate. As there is no single statistical method that can account for all of these aspects of the dataset, our approach was to use a series of statistical techniques, each with its own limitations, to build a synthetic understanding of the controls on AGB.

We first investigate the continuous variables, presenting Spearman's correlation coefficients, accounting for spatial autocorrelation using Dutilleul's method [32]. For categorical soil variables, we use ANOVA to assess their potential impacts on response variables. We then take an information-theoretic approach, testing all possible combinations of the climate, fragmentation and soil variables, selecting the best model on the basis of the lowest Akaike's information criterion, corrected for finite sample sizes (AIC_C_). We assume all of the ordinary least-squares (OLS) models within two AIC_C_ units of the lowest AIC_C_ model are plausible alternatives in terms of explaining variation in the dataset [33,34]. Extensive preliminary analysis showed which pairs of variables had the most explanatory power *T*_min_ or *T*_COLDQ_, *T*_max_ or *T*_WARMQ_, *P*_min_ or *P*_DRYQ_, *P*_max_ or *P*_WETQ_. We selected *T*_min_, *T*_WARMQ_, *P*_min_ and *P*_WETQ_ for inclusion in the models to better allow comparisons of models across response variables. Following this, the low AIC_C_ models were checked for parameter redundancy by removing redundant variables that are the same sign (i.e. if *T*_A_ and *T*_WARMQ_ are included and of the same sign, then one is removed based on importance values), and the full suite of models was run again, minus these redundant terms (see the electronic supplementary material for further details). Removing redundant terms aids the interpretation of the results and avoids the possible problem of over-fitting sometimes associated with larger datasets [34].

We then account for spatial autocorrelation in our OLS models. As there is no definitive technique to account for spatial autocorrelation [35], we follow the recent example of Quesada *et al.* [3] who used eigenvector-based spatial filtering (extracted by principle component of neighbour matrices [36,37]) on a similar dataset from Amazonia, which aides cross-continental comparisons. We identify the spatial filters significantly correlated with the residuals from the OLS model, and re-run the identical explanatory variables as in the OLS model plus the selected filters, termed spatial eigenvector mapping (SEVM) models. We computed other less stringent filtering methods, but as these inform more on the underlying structure of the variables rather than addressing our specific hypotheses we omit them for brevity (see [3]). We used spatial ecology in macroecology, version 4.0 [37] for the analysis.

## 3. Results

### (a) General patterns

**Figure 2.**
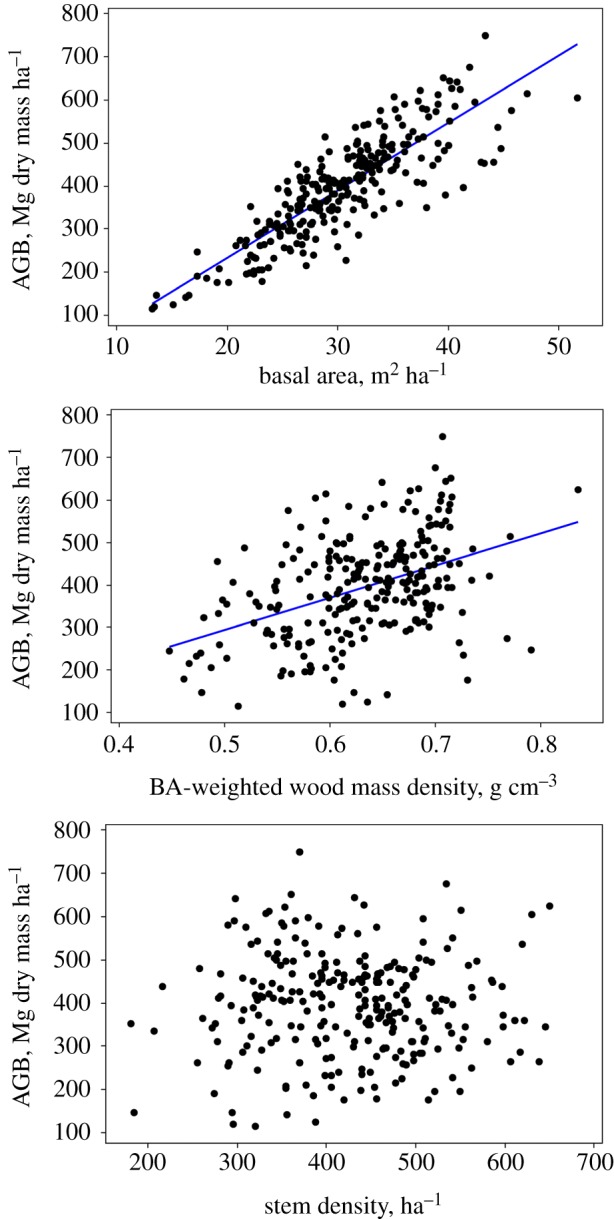
Above-ground biomass (AGB) plotted against basal area, basal area-weighted wood mass density, and stem density for 260 plots in closed-canopy tropical forest. OLS lines are, AGB = −78.6 + 15.6 × BA (*r*^2^ = 0.71); AGB = −82.4 + 755 × WMD_BA_ (*r*^2^ = 0.18). (Online version in colour.)

**Figure 3.**
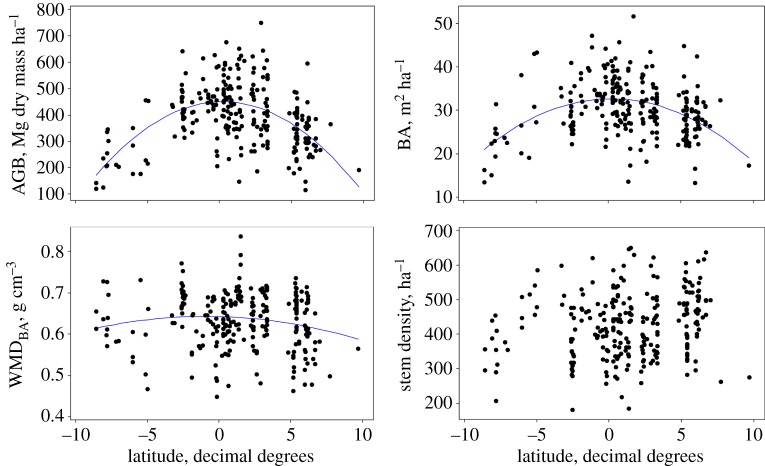
Above-ground biomass (AGB), basal area (BA), basal area-weighted wood mass density (WMD_BA_), and stem density for 260 plots versus latitude in decimal degrees. Quadratic fits are AGB = 451.6–3.57 × latitude^2^ (*r*^2^ = 0.31, *p* < 0.001); BA = 32.7–0.150 × latitude^2^ (*r*^2^ = 0.18, *p* < 0.001); WMD_BA_ = 0.641–0.00051 × latitude^2^ (*r*^2^ = 0.02, *p* = 0.02). (Online version in colour.)

The mean stem density of the 260 plots was 425.6 stems ha^−1^ greater than or equal to 100 mm diameter (95% CI: ±11.1; figure 1). The mean BA was 30.3 m^2^ ha^−1^ (CI: ±0.77; figure 1). The mean WMD was 0.648 g cm^−3^ (CI: ±0.0063) on a stems basis, with WMD_BA_ (BA-weighted WMD) being 0.633 g cm^−3^ (CI: ±0.0080). The mean above-ground live biomass was estimated at 395.7 Mg dry mass ha^−1^ (CI: ±14.3; figure 1). The relationships between AGB and three possible proximate causes of variation, stems ha^−1^, BA and WMD_BA_ differ from strong (BA) to non-significant (stems ha^−1^; figure 2). There was a strong significant convex relationship of AGB with latitude (*p* < 0.001), with AGB tending to be greatest near the equator, alongside more moderate significant relationships with BA and WMD_BA_ (*p* < 0.001 and *p* = 0.02), but not for the number of trees per hectare (figure 3). Quadratic fits thus suggest that, on average, forests on the equator have high AGB (452 Mg dry mass ha^−1^), relatively high BA (32.7 m^2^ ha^−1^), and relatively high WMD_BA_ (0.64 g cm^−3^; figure 3). Surprisingly, *T*_A_ does not show a clear convex relationship with latitude (see the electronic supplementary material). Counterintuitively, many lower latitude plots have lower temperatures because they are at a higher altitude. Similarly, there is no latitudinal relationship with *P*_A_. This is because *P*_DRYQ_ is convexly related to latitude, whereas *P*_WETQ_ is concavely related, obviating any latitudinal trend in *P*_A_ (see the electronic supplementary material). Average soil development age also peaks at the equator, where heavily weathered ferralsols dominate, as does fragment size and distance to the nearest clearing. These correlations imply that lower *T*_A_, consistent moderately high *P*_A_, a lack of habitat fragmentation, and attributes associated with highly weathered soils may promote the highest AGB. The values for all plots are provided in the electronic supplementary material.

The different forest types had different AGB and other structural parameters. The five swamp locations had lower AGB, 322.2 Mg dry mass ha^−1^ (not significantly so, *p* = 0.16), and significantly lower BA (24.2 m^2^; *p* = 0.03) than the *terra firme* plots. This was attributable to fewer large diameter stems in such forests, as the total number of stems was not lower (428 ha^−1^) and WMD_BA_ was much higher than for the non-swamp plots (0.728 g cm^−1^). These data confirm the outlier status of the swamp plots, which were therefore excluded from the final information-theoretic analysis. Monodominant forests, dominated by *Gilbertiodendron dewevrei*, are a common occurrence in Central Africa (*n* = 23) and were found to have significantly higher AGB than non-*Gilbertiodendron*-dominated forests (514.9 versus 384.1 Mg dry mass ha^−1^; ANOVA, *p* < 0.001), but not BA (32.2 versus 30.2 m^2^). They also had significantly lower stem density (340 versus 434 stems ha^−1^; *p* < 0.001) and significantly higher WMD_BA_ (0.696 versus 0.627 g cm^−3^; *p* < 0.001).

### (b) Relationships with single variables

**Figure 4.**
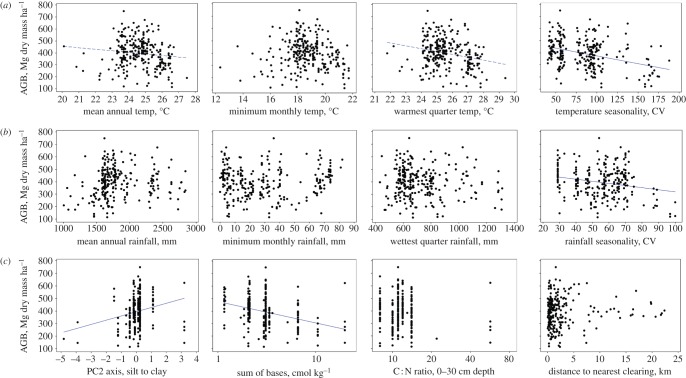
Bivariate plots of AGB and (*a*) temperature (top; mean annual temperature, temperature coldest month, temperature in warmest quarter, temperature of coefficient variation, left to right), (*b*) rainfall (middle; mean annual rainfall, rainfall in driest month, rainfall wettest quarter, rainfall coefficient of variation, left to right) and (*c*) soil and fragmentation (bottom; PCA axis two, silt to clay texture, sum of bases in topsoil, carbon to nitrogen ratio in topsoil, distance to nearest forest edge and clearing, left to right; note log scale). Dashed regression lines indicate a significant relationship before accounting for spatial autocorrelation, solid lines after accounting for spatial autocorrelation (full details and equivalent graphs for BA, WMD_BA_ and stem density in electronic supplementary material). CV is coefficient of variation. (Online version in colour.)

AGB was found to be positively spatially autocorrelated over distances to approximately 700 km, with similar values for BA (approx. 500 km), and less for WMD_BA_ (approx. 300 km), but no clear pattern for stem density (see the electronic supplementary material). Considering bivariate relationships first, although the signs of the AGB relationships with *P*_A_, *P*_min_ (positive), *P*_WET_ and *P*_CV_ (negative), and all temperature variables (negative) were as predicted, only *T*_CV_ and *P*_CV_ were significantly negatively correlated with AGB after adjustment of the effective degrees of freedom to account for spatial autocorrelation (figure 4). The soil variable ∑B was, however, significantly negatively correlated with AGB, and PC2 (clay) significantly positively correlated, even after accounting for spatial autocorrelation (figure 4). The results for BA show significant negative relationships with only *T*_A_ and *T*_WARMQ_ (after accounting for spatial autocorrelation), although ∑B was marginally significant (*p* = 0.06). For WMD_BA_, only PC2 (clay) was significantly related, suggesting clay-rich soils have higher WMD_BA_ than silt-rich soils. Note that the ∑B and C : N correlations are strongly influenced by the histosol soils which often occur beneath swamps. For stem density, none of the studied variables was found to be significantly correlated after accounting for spatial autocorrelation. No edge or fragment size variables were significantly correlated with AGB, BA, WMD_BA_ or stem density. Correlation coefficients before and after accounting for spatial autocorrelation plus bivariate plots are in the electronic supplementary material.

The 260 plots were located on 17 major soil types, within eight major classes. The most common soil class was ferralsols (*n* = 94), and most common type orthic ferralsols (*n* = 74). An ANOVA on the plots overlying common soil classes (*n* ≥ 5 plots) showed that AGB on cambisols, nitosols and acrisols (373, 358 and 320 Mg ha^−1^, respectively) was significantly lower than that on ferralsols and arenosols (436 and 444 Mg ha^−1^ respectively; see electronic supplementary material for full results). That is, the relatively fertile and developmentally younger soils had lower AGB than either the sandier and lower fertility arenosols, or deeply weathered but nutrient-poor ferralsols. For BA, the only significant difference was the lower values on acrisols (27.5 m^2^ ha^−1^) compared with ferralsols (32.0 m^2^ ha^−1^). The plots on arenosols, cambisols and nitosols all had similar BA (30.7, 30.3, 30.2 m^2^ ha^−1^, respectively). Developmentally younger and relatively fertile acrisols and cambisols have significantly lower WMD_BA_ (0.609 and 0.617 g cm^−3^) than arenosols (0.660 g cm^−3^) or histosols, which at 0.728 g cm^−3^ were significantly higher than all other soil classes. For stem density, nitosols were significantly higher (477 stems ha^−1^) than either ferralsols or arenosols (423 and 395 stems ha^−1^, respectively). Analysis of soil types showed similar results to the soil class ANOVAs. For example, developmentally younger soils had lower AGB, with xanthic ferralsols having the highest AGB (463 Mg ha^−1^), double that of the lowest class (chromic cambisols, 232 Mg ha^−1^). Of three within-soil class comparisons (e.g. ferric versus orthic acrisols), the more fertile soil type had lower AGB in each case. All ANOVA results are in the electronic supplementary material.

### (c) Relationships considering all variables

The lowest AIC_C_ OLS model for AGB included *P*_A_, *P*_WETQ_, *T*_A_, *T*_WARMQ_, C : N, ∑B and PC2 (silt–clay continuum) soil variables and explained 32.4% of the variation in the dataset (table 1). *P*_A_ was positively related to AGB, higher by 1.3 Mg dry mass ha^−1^ for each 10 mm increment of rainfall, unless precipitation in the wettest quarter was higher, when this would reduce AGB. Put another way, precipitation in the nine drier months is positively related to AGB, whereas it is negatively related in the wettest three months. Similarly, *T*_A_ was positively related to AGB and *T*_WARMQ_ negatively related. Taken together, this implies a net AGB difference of approximately −11.7 Mg dry mass ha^−1^ (approx. 3% of AGB) for each degree Celsius of higher temperature. C : N ratio was negatively related to AGB, i.e. higher phosphorous availability is related to higher AGB (if the assumption that C : N is a surrogate for plant available phosphorus, as we argue in the methods, holds). Conversely, higher ∑B was negatively related to AGB; clay-rich soils (PC2) were positively related. Standardized regression coefficients show that soil and temperature effects are larger than the precipitation effects. There were 10 other models within two AIC_C_ units, and therefore plausible, with each model removing one or more of ∑B, C : N and *P*_WETQ_, and/or adding a negative *F*_E_ term (i.e. lower AGB farther from edges). Overall, there are opposing sign temperature (*T*_A_, *T*_WARMQ_), precipitation (*P*_A_, *P*_WETQ_) and soil fertility (C : N, ∑B) terms affecting forest AGB. The models’ did not show strong spatial structure (see the electronic supplementary material). Excluding the 23 *Gilberiodendron*-dominated plots does not alter any conclusions.

**Table 1.** Lowest AIC_C_ model fits (*β*) and *p*-values for above-ground biomass (AGB), basal area (BA), BA-weighted wood mass density (WMD_BA_) and stem density (S_D_) without (OLS) and with (SEVM) spatial filters to account for the spatial structure in the dataset. *T*_A_, average mean annual temperature; *T*_min_, minimum monthly temperature; *T*_WARMQ_, warmest quarter temperature; *P*_A_, precipitation per annum; *P*_min_, precipitation in the driest month; *P*_WETQ_, precipitation in the wettest quarter; C : N, carbon: nitrogen ratio; ∑B, sum of bases; principal components analysis first two axes on soil structure, PC1 relating to sand content, and PC2 clay–silt content; *F*_E_, distance to the nearest forest edge; *F*_A_, area of forest fragment (numbering of the spatial filters is inconsequential).

variable	AGB, OLS model		AGB, SEVM model		BA, OLS model		BA, SEVM model		WMD_BA, OLS model		WMD_BA, SEVM model		stem density, OLS model		stem density, SEVM model	
	**β**	*p*	**β**	*p*	*B*	*p*	**β**	*p*	**β**	*p*	**β**	*p*	**β**	*p*	**β**	*p*
constant	766.54	<0.001	332.65	<0.001	64.12	<0.001	54.17	<0.001	0.42854	<0.001	0.44157	<0.001	634.47	<0.001	648.59	<0.001
*P*_A_	0.13	0.005	0.16	<0.001	0.006	0.002										
*P*_min_									0.00061	0.002						
*P*_WETQ_	−0.17	0.062	−0.33	<0.001	−0.007	0.065							0.091	0.006		
*T*_A_	79.16	0.002							0.01007	0.008			−10.84	0.047	−8.75	0.09
*T*_min_											0.01021	<0.001				
*T*_WARMQ_	−90.83	<0.001			−1.54	<0.001	−0.908	0.017								
C : N ratio	−7.04	0.08							−0.00599	0.029						
∑B	−6.87	0.11														
PC1 sand									−0.00465	0.011						
PC2 clay	20.68	0.043	14.20	0.122	1.95	<0.001	1.32	0.013	0.01136	0.045	0.00943	0.09	11.78	0.142		
*F*_E_					−0.15	0.07	−0.20	0.016					−2.52	0.06	−2.22	0.085
*F*_A_									−0.00011	0.079	−0.00013	0.033				
filter no. 1			607.76	<0.001			14.62	0.021								
filter no. 2			483.37	<0.001			13.17	0.058			0.23125	<0.001				
filter no. 3															−406.32	<0.001
filter no. 4			−260.81	0.043			−21.53	<0.001								
filter no. 6											−0.17782	0.003				
filter no. 7											0.16751	0.005			−197.36	0.024
filter no. 10											0.16751	0.005				
filter no. 12															185.26	0.033

The lowest AIC_C_ model after applying the SEVM filters was similar to the OLS models, including *P*_A_, *P*_WETQ_ and PC2 but no longer with any temperature or soil fertility variables (table 1). Eight other low AIC_C_ models were identified as plausible: these were without PC2 (five models), or added ∑B or C : N in some combination, in common with the OLS models. In one model, temperature terms are retained, but these are net positive. Thus, the main impact of the filters was to remove the overall negative temperature effect. However, this result should be treated cautiously, because the SEVM residuals models are very similar to those from the OLS models (see the electronic supplementary material).

Given the importance of high temperature impacts for the future of tropical forests as well as the ambiguity of the results, we re-ran the models including only the warmest forests: those plots less than 500 m. All the low AIC_C_ OLS models again included a negative relation with temperature, as did 10 of 11 low AIC_C_ SEVM models. Overall, among the warmest African forests, if temperature variation has an impact on AGB variation, then it is negative.

The lowest AIC_C_ OLS model for BA was similar to the AGB OLS models, but with the two soil fertility terms not included, and an added negative *F*_E_ term; this model explained 24.6% of the variation in the dataset (table 1). Twelve other low AIC_C_ models were identified, adding to the best model negative ∑B and/or C : N terms, adding a positive *T*_A_ term or removing *P*_WETQ_ or *F*_E_ in some combination. Thus, the low AIC_C_ BA and AGB models were collectively similar. Adding the SEVM filters retained similar results, but removed the precipitation terms and reduced the magnitude of both the negative *T*_WARMQ_ and positive PC2 terms (table 2). The five alternative low AIC_C_ models include the missing *P*_A_ and *P*_WETQ_ terms and/or the C : N term. Hence, for BA, the temperature relationship is negative and larger than that for AGB (approx. 3–5% lower BA in forests growing under higher air temperature). The spatial residuals were improved using the SEVM filters over the OLS models.

**Table 2.** Mean environmental parameters for forest inventory plots in West, Central and East Africa. Alt, altitude; *T*_A_, mean annual temperature; *T*_min_, minimum monthly temperature; *T*_WARMQ_, warmest quarter temperature; *T*_CV_, temperature coefficient of variation; *P*_A_, precipitation per annum; *P*_min_, precipitation in the driest month; *P*_WETQ_, precipitation in the wettest quarter; *P*_CV_, precipitation coefficient of variation; *F*_E_, distance to the nearest forest edge; *S*_DEV_, soil development index; sand, % sand in topsoil; ∑B, sum of bases; AGB, above-ground biomass; BA, basal area; WMD, wood mass density; WMD_BA_, basal area-weighted WMD; SD, stem density. Different letters indicate significant differences following Tukey's tests following ANOVA.

Region	*n*	Alt	*T*_A_	*T*_min_	*T*_WARMQ_	*T*_CV_^c^	*P*_A_	*P*_min_	*P*_WETQ_	*P*_CV_^c^	*F*_E_^a^	*S*_DEV_^c^	Sand^b^	∑B^b^	C : N^b^	AGB	BA	WMD	WMD_BA_	SD
		m	°C	°C	°C		mm	mm	mm		Km		%	cmol/kg		Mg ha^−1^	m^2^ ha^−1^	g cm^−3^	g cm^−3^	ha^−1^
West	42	171 A	26.0 A	20.6 A	27.3 A	98 A	1891 A	39 A	721 A	51 A	1.2 A	2.1 AB	55 A	3.3 A	13.4 A	305 A	26.4 A	0.64 A	0.61 A	433 A
Central	196	461 B	24.5 B	18.6 B	25.3 B	73 B	1850 A	28 B	739 A	54 A	3.5 B	2.3 A	52 A	3.4 A	11.1 B	429 B	31.5 B	0.65 A	0.64 B	425 A
East	22	599 C	23.5 C	16.1 C	25.3 B	157 C	1374 B	23 B	684 A	72 B	0.9 A	1.7 B	44 A	6.6 B	10.0 C	274 A	27.4 A	0.61 B	0.61 A	423 A

^a^*n* = 230 (owing to missing values due to clouds in satellite imagery).

^b^Excluding five histosols in Central Africa.

^c^Arbitrary units; higher values denote more variable conditions for climate parameters, and denote more developed and less fertile conditions for soils.

The lowest AIC_C_ OLS model for WMD_BA_ included positive effects of *P*_min_, positive *T*_A_ impact, positive PC2 (clay) plus negative C : N relationship, PC1 (sand) and *F*_A_ terms. The model explained 15.0% of the variation in the dataset (table 1). There were three alternative low AIC_C_ models, involving an additional negative term ∑B, negative *T*_min_ or without the *F*_A_ term, respectively. The lowest AIC_C_ SEVM model retained only a strong positive relationship with temperature, PC2 and the negative *F*_A_ terms. Seven alternative low AIC_C_ models included an additional PC1, C : N, and/or *T*_A_ term or dropped PC2 in various combinations. Overall, there is a strong increase in WMD_BA_ with higher air temperature, a likely decrease with C : N, and an increase in sandy- or clay-rich soils. The precipitation and fragmentation terms are weak in comparison with the temperature and soil effects. The spatial residuals were improved over short distances when using the SEVM filters.

The lowest AIC_C_ OLS model explained only 7.1% of the variation in stems ha^−1^; the model included a positive relationship with *P*_WETQ_ and PC2, a stronger negative *T*_A_ term and a negative *F*_E_ term (i.e. more stems closer to forest edges; table 1). The 12 alternative low AIC_C_ models differed from the other dependent variables analysed, as models of stem density ranged from those including only single variables (either *P*_WETQ_ or *T*_A_) to including all six parameters (*P*_WETQ_, *T*_A_, PC2, ∑B, C : N, *F*_E_). Terms for ∑B and C : N were positive and were each included in four of 13 models. The SEVM low AIC_C_ model were similar, with 20 selected, again spanning models including from one to six environmental parameters. Only the negative *T*_A_ and *F*_E_ terms were retained in the SEVM lowest AIC_C_ model (table 1).

### (d) Comparing West, East and Central African forests

The main AGB, BA, WMD_BA_ and stem density results are replicated when only plots from Central Africa, the largest regional group of plots, are used in the analyses. The environment–structure relationships reported above are thus not driven by combining plots from within the West, Central and East Africa regions. There are however systematic differences among the regions. Although plots grouped into West, Central and East African regions showed no differences in mean stem density, those sampled in Central African forests had over one-third higher AGB than either the West or East African forests (table 2). AGB differences among forests are partly caused by BA differences, which largely mirror AGB, with WMD_BA_ variations also being important, with this stand-level trait being significantly higher for Central African forests than their West or East African counterparts. By contrast, WMD was not significantly different between West and Central Africa, whereas WMD in the sampled East African forests remained significantly lower. Thus, the sampled West African forests are characterized by relatively low AGB, caused by low BA and lower WMD of larger trees, whereas the sampled East African forests are characterized by even lower AGB, but driven by low BA, and lower WMD of all size classes of trees. In terms of the environment, the sampled West African plots are in forests that tend to be warmer and more fragmented, and have higher C : N ratio (lower phosphorus) compared with Central Africa. The sampled East African forests, by contrast, are cooler, drier, more fragmented, and on developmentally younger and higher ∑B and lower C : N ratio soils than Central African forests, suggesting multiple combinations of variables may lead to low AGB forests (table 2).

## 4. Discussion

African tropical forests are characterized by relatively high AGB, at 395.7 Mg dry mass ha^−1^, which in Central Africa—where the majority of the areal extent of African closed-canopy forest is located—is higher at 429 Mg dry mass ha^−1^, and statistically indistinguishable from the high AGB stocks of the forests of Borneo at approximately 445 Mg dry mass ha^−1^ [4]. These African and Asian values are significantly higher than forest AGB reported from a synthesis across Amazonia at 289 Mg dry mass ha^−1^ [1]. These results show that there is a difference between generally higher AGB palaeotropical forest versus generally lower AGB neotropical forest, which supports recent studies showing neo- versus palaeotropical differences in stem allometry, BA [6] and AGB [7] based on more limited African data (summarized in table 3). However, all such results should be treated cautiously because of a fundamental limitation: we are never measuring AGB directly, but are rather estimating it using imperfect allometric relationships. Improved allometric relationships (increased sample sizes of trees of known mass; better characterization of height–diameter relations [39]; more WMD measurements) plus more extensive sampling of tropical forests will help refine future estimates.

**Table 3.** Cross-continental comparisons of forest structure from networks of intact old-growth closed-canopy tropical forest for the largest biogeographic regions from Africa, Asia and the Americas.

parameter	Central Africa	Borneo, Asia	central/east Amazonia
above-ground biomass, Mg dry mass ha^−1^	429^a^	445^b^	341^c^
basal area, m^2^ ha^−1^	31.5^a^	37.1^b^	29.0^c^
wood mass density, g cm^−3^	0.65^a^	0.60^b^	0.66^c^
stem density, ≥100 mm diameter, ha^−1^	425^a^	602^b^	597^d^
mean tree size, m^2^	0.074	0.062	0.049
mean tree height, stem 100 mm diameter, m	13.3^e^	11.9^e^	10.6^e^
mean tree height, stem 400 mm diameter, m	30.8^e^	30.3^e^	26.1^e^
mean tree height, stem 1000 mm diameter, m	43.5^e^	46.0^e^	39.0^e^

^a^This study.

^b^From [4].

^c^From [22].

^d^From [38].

^e^From [7].

The high AGB in African forests is coupled with a very low stem density, 426 stems greater than or equal to 100 mm ha^−1^, compared with 602 ha^−1^ in Borneo [4] and 592 in Amazonia [38]. Low stem density is therefore the signature structural feature of African tropical forest compared with other continents. It then follows that mean tree size is greater in Africa than elsewhere in the tropics (table 3). WMD in Africa (0.65 g cm^−3^) is similar to that in central and eastern Amazonia (0.66 g cm^−3^; [22] but higher than forests in Borneo (0.60 g cm^−3^; [4] or western Amazonia (0.56 g cm^−3^; [22]). This result points towards African forests being dominated by relatively low-frequency disturbance regimes over at least recent decades allowing trees time to grow large and stands to self-thin. This point is reinforced by the relatively common occurrence in Central Africa of monodominant stands, dominated by a single tree species (e.g. *Gilbertiodendron dewevrei, Cynometra alexandri*), compared with the rarity of monodominance in Amazonia or Southeast Asia [40]. These stands, which can cover tens to thousands of hectares, lack obvious edaphic or climatic controls, occur instead in areas that appear to lack disturbance over the long term [40–43]. The even lower stem density, higher AGB and higher WMD_BA_ and slower dynamics of these forests, compared with nearby mixed-species stands, provides further support for this view [40–43]. On the other hand, the extremely low stem density in African forests may relate to the very high large animal biomass: elephants (*Loxodonta africana cyclotis*), gorillas (*Gorilla gorilla gorilla*) and other large herbivores such as bongos (*Tragelaphus eurycerus*) may keep the density of small trees very low [44]. This view is reinforced by a recent paper from Southeast Asia showing a large increase in sapling density when the large animal fauna is extirpated [45].

Our results, in conjunction with recent studies across Borneo [4] and Amazonia [2,3] and pan-tropical analyses [6,7], thus provide some evidence that the three major continental groupings of tropical forest differ in their basic structural parameters, with African forests being tall stature, high AGB, low stem density and high WMD; Borneo characterized by tall stature, high AGB, high stem density and lower WMD, and Amazonian stands associated with shorter stature, lower AGB, high stem density and across most of Amazonia high WMD (table 3). The implication is that there are either (i) major cross-continental allocation differences or (ii) NPP is greater across the palaeotropics, or (iii) biomass residence times are longer (i.e. disturbance rates are lower) in the palaeotropics. The low stem density in African forests points towards Amazon–Africa differences being more likely a result of different biomass residence times, with Africa–Borneo differences being more likely based on NPP differences (high AGB, but not low stem density, and low WMD_BA_ suggesting higher NPP). A recent pan-tropical analysis of biomass residence times is consistent with these conclusions despite few data from the palaeotropics [31]. Alternatively, the differences may relate to the history and biogeography of the different regions, particularly the dominance of the Dipterocarpaceae across Southeast Asia.

Spatially, our results show clear patterns such as the relationship with latitude, with the highest AGB forests near the equator. Here, we briefly consider the impact of soil parameters, rainfall, temperature and forest fragmentation, in turn, followed by conclusions on the possible causes of difference among the sampled plots in Central, West and East Africa.

The soil data derive from a gridded global database rather than from the plots themselves and thus must be treated cautiously. Furthermore, the analyses were sensitive to outlier soil types (leading to the exclusion of swamp plots on histosols from the latter analyses). The AGB–soil fertility results were, however, partially consistent with both our stated hypotheses. First, we hypothesized that higher resource availability increases NPP increasing AGB. Higher C : N ratios were associated with lower AGB; and because C : N is negatively related to total extractable phosphorus [5], this implies that it might be higher phosphorus availability that is associated with higher AGB. This accords with studies that show that phosphorus can limit tree growth in tropical forests, and consistent with those from Amazonia, where AGB is positively linked with total soil phosphorus (see [3] and references therein). Second, and counter to this, faster-growing forest stands may become dominated by low WMD species with shorter lifespans (lower **τ**_W_), and hence lower AGB. Consistent with this, when ∑B was included in low AIC_C_ models, it was strongly negatively associated with AGB. Again, this is accords with results from Amazonia where AGB is negatively related to exchangeable soil potassium [3]. However, considering WMD_BA_, the results are not as clearly interpretable, as the lowest AIC_C_ SEVM model includes no soil fertility terms. While some alternative models do include negative ∑B terms, when included C : N terms imply a positive phosphorus–WMD_BA_ relationship, counter to predictions. Our working hypothesis to account for these results is that a greater supply of limiting nutrients leads to higher AGB, because higher NPP levels more than offset any lowering of WMD_BA_ and thereby **τ**_W_, whereas greater supplies of non-limiting nutrients lead to lower AGB, because **τ**_W_ is lower and NPP is not increased. The data on soil physical variables are too limited to make robust deductions, as soil depth and other physical conditions remain unknown. AGB was, however, positively associated with developmentally older soils, and with clay-rich soils compared with silt-rich soils (PC2), suggesting that deep well-structured clay-rich soils may be of benefit to trees in attaining a large size. Interestingly, the PC2 term was usually a stronger term in the analyses suggesting impacts on *τ*_W_ may be a more important driver of differences in AGB, BA and WMD than soil fertility terms. *In situ* sampling is required to elucidate the impacts of the physical and chemical characteristics of soils on AGB and its component drivers.

Biomass relationships with rainfall were likewise broadly consistent with *a priori* expectations. In all OLS and SEVM analyses, the low AIC_C_ models included terms in which higher rainfall outside of the wettest quarter increased AGB, implying increased NPP owing to higher water availability. The results are broadly consistent with those from Amazonia where precipitation in the dry season is positively associated with variation in AGB [3], and across Borneo where *P*_A_ is positively associated with AGB [4], and wider syntheses [46]. Our results differ from some previous reports in that more rainfall in the wettest part of the year was correlated with lower AGB. However, our results are consistent with the limited data showing than ever-wet forests tend to have lower NPP [14,16,17] and AGB [15]. This implies that the excess rainfall either reduces NPP (owing to more clouds, or perhaps soil saturation effects) or elevates mortality, thereby shortening AGB residence times.

The results of the possible impact of the temperature-related variables on AGB were complex. Bivariate plots and the low AIC_C_ OLS models both showed that high *T*_WARMQ_ was associated with low AGB. By contrast, only one of eight SEVM low AIC_C_ models included a negative net temperature term. This suggests that after accounting for the spatial structure in the temperature data the negative effect of temperature is removed (but note that the SEVM filters did not substantially improve the residuals in the model, see electronic supplementary material). The cause of the difference is due to filter 1 in the SEVM analyses, which is deeply concave with distance. This is driven by a preponderance of higher elevation plot locations around the eastern and western periphery of the Congo Basin, giving long-distance temperature symmetry in the dataset. Thus, when plots from only Central Africa are retained the same shaped SEVM filter 1 is retained, whereas when only plots less than or equal to 500 m are retained in the analysis (i.e. higher altitude east and west Central Africa region plots are removed), the negative temperature effect from the OLS model is retained in most low AIC_C_ models. A negative relationship between temperature and AGB could arise through a variety of mechanisms (e.g. higher respiration costs; midday declines in photosynthesis [19]) and is consistent with a demonstrated negative relationship of *T*_A_ with wood productivity in Amazonia [3]. Such temperature effects have not, in general, been detected in the past [3,4,46], but it is worth noting that previous AGB studies have analysed smaller sample sizes than in this study.

The lowest AIC_C_ OLS model predicts that forests have 11.7 Mg dry mass ha^−1^ lower AGB for each higher degree of temperature (3% of AGB). Recent model results give divergent projections of the magnitude of temperature impacts on tropical vegetation biomass. For example, our results are about 20–40% of the impact predicted by one recent model [47]. However, a more recent result suggests that approximately 8 Mg C ha^−1^ is lost at equilibrium per degree of warming from the tropical land surface, of which about half is related to vegetation (and half to soils; [48] and P. Cox 2013, personal communication). Thus, assuming biomass is approximately 50% carbon, and 75% of this vegetation biomass is above-ground, the model-predicted difference is approximately 6 Mg dry mass ha^−1^ for AGB across all tropical vegetation types. Thus, our results appear, given our focus on forests with high AGB, broadly similar to the model results in [48].

Considered another way, if we substitute space for time, and assume that air temperature is rising by 0.26°C per decade [18], this would equate to a loss of approximately 0.3 Mg dry mass ha^−1^ yr^−1^ for contemporary forests (0.08% of AGB). Such a decline has not been detected in African forests, indeed, a much larger increase of 1.2 Mg dry mass ha^−1^ yr^−1^ has been documented [26]. This is has been attributed, in part, to higher atmospheric CO_2_ concentrations, an interpretation consistent with theory and model results [49] and the observation that increasing forest AGB is a general, long-term and global phenomena [50]. Thus, if there is a negative impact of temperature on tropical AGB currently, then it is being overwhelmed by other positive effects such as increasing CO_2_. If CO_2_ effects saturate in the future, then any negative impact of temperature should become apparent.

A further surprising temperature effect was the strong positive relationship of WMD_BA_ with *T*_A_ (table 1). For each higher degree, WMD_BA_ increases by 0.01 g cm^−3^ (approx. 1.5%). Combining this with the WMD_BA_–AGB relationship in figure 1 suggests each higher degree increases AGB by 7.6 Mg dry mass ha^−1^ purely related to higher wood density in these forests. The same strong positive temperature–wood density relationship is shown across Amazonia [3,51] and larger-scale analyses across the Americas [52] and China [53]. The positive WMD–*T*_A_ relationship is thought possibly to be a necessary adaptation to the effect of increases in temperature reducing the viscosity of water [54] and the generally higher vapour pressure deficits encountered by trees living in warmer climates, which, all things being equal, may benefit higher WMD trees as they tend to have increased drought tolerances. This effect has been shown in experiments [55]. Thus, in terms of AGB, the strong negative BA–temperature relationship is somewhat offset by the positive WMD–temperature. Additionally, in global change terms, hypothesized decreases in WMD of forest stands caused by better conditions for growth [26] may be somewhat offset by the increase in WMD from higher air temperatures.

The habitat fragmentation results are a difficult to interpret. This may be related to the relatively weak indices derived for distance from the nearest edge and fragment area. Reduced BA and lower stem density further from edges could be related to a lower density of elephants and other large herbivores, and the known thickening of vegetation very close to forest edges. However, the lower WMD_BA_ in larger fragments does not fit this pattern. Much finer scale analyses with better metrics of distance from edges, including different types of edge [56], will be necessary to ascertain the true effects of fragmentation on forest biomass. More generally, the stem density models explained a much lower proportion of the variation in the data (7%) compared with the AGB, BA and WMD_BA_ models. The large number of low AIC_C_ models and their very different structure suggest that stem density is not primarily controlled by the factors we measured. However, there was a strong impact of temperature, with each greater degree Celsius associated with 10 stems fewer per hectare. We know of no reason for such a relationship. Given that the plots were selected as ‘old-growth’, and density is uniformly low across the continent, this suggests that stem density is primarily an emergent property of the long-term disturbance regime, and this has been relatively low across the African tropical forests over recent decades.

We suggest that the lower AGB in West African forests compared with Central African forests is likely to be caused by a complex mix of factors. First, the low WMD_BA_ of the West African forest, but not WMD, compared with Central African forest, suggests a species composition difference, with large trees having lower WMD in West Africa. This may be caused by the removal of elephant populations over the past few hundred years, and a generally more depauperate fauna, leading to a lack of dispersal of larger seeded species that tend to be associated with higher WMD. Second, the two key environmental differences that may account for the lower West African AGB are the high C : N ratio (likely associated lower phosphorus levels), and higher average air temperatures. By contrast, the lower AGB in forests of East Africa appears to be related to developmentally younger soils, with high ∑B, and therefore lower WMD for all size classes of stems. This is reinforced by the evidence of the relatively low stature of East African forests, with trees being significantly shorter than elsewhere in Africa [7,39,57]. Differences in forest structure correlated with soil age from central to eastern Africa may be similar to the east–west Amazon differences related to soil development age; if so, then we would expect to see similarly high stem turnover and shorter **τ**_W_ in East compared with Central Africa when recensuses of these inventory plots are completed. While both East and West Africa are also more fragmented than Central Africa, our OLS results do not point to this being a major factor in their lower AGB. However, our findings clearly show that there are multiple combinations of environmental conditions that lead to low AGB.

Overall, our results, combined with others, suggest pan-tropical AGB–environment consistencies. These have potential implications for the future behaviour of tropical forests within the changing Earth system. While space for time substitutions must be treated with caution, especially in the light of the inevitable spatial and temporal autocorrelations, the results suggest that the physiological effects of higher air temperature may to some degree offset ongoing increases in AGB expected to flow on from NPP enhancements associated with increased atmospheric CO_2_ concentrations (as models show [43,58]). Perhaps more importantly, the influence of rainfall may be large but difficult to quantify, with precipitation in the driest nine months is positively related to AGB, whereas precipitation in the wettest three months is negatively associated with AGB. This potential future change appears underappreciated by the global change community, which has focused significant attention on the impacts of droughts [59], but not the implications for forests of wet-season rainfall increases. Higher temperature and concomitant decreases in water viscosity will also probably lead to a shift towards higher WMD species, countering any shift to lower WMD species from either increasing forest dynamism [60,61], or from growth increases from higher resource availability which have been hypothesized to benefit lower WMD species [26,38,49]. Such conclusions are necessarily tentative, because the underlying NPP and biomass residence time parameters need to be analysed across the environmental space that tropical forests occur to more robustly test for possible generalizations. Once identified, such patterns and processes can then be incorporated into predictive models of the future. This will be possible if emerging pan-tropical networks are well-distributed, long-term, and efforts are made to ensure that monitoring sites incorporate site-specific soil analyses and local climate data.
